# Selict-seq profiles genome-wide off-target effects in adenosine base editing

**DOI:** 10.1093/nar/gkaf281

**Published:** 2025-04-10

**Authors:** Kexin Yuan, Xin Xi, Shaoqing Han, Jingyu Han, Bin Zhao, Qi Wei, Xiang Zhou

**Affiliations:** College of Chemistry and Molecular Sciences, Wuhan University, Wuhan, Hubei 430072, PR China; College of Chemistry and Molecular Sciences, Wuhan University, Wuhan, Hubei 430072, PR China; College of Chemistry and Molecular Sciences, Wuhan University, Wuhan, Hubei 430072, PR China; College of Chemistry and Molecular Sciences, Wuhan University, Wuhan, Hubei 430072, PR China; College of Chemistry and Molecular Sciences, Wuhan University, Wuhan, Hubei 430072, PR China; College of Chemistry and Molecular Sciences, Wuhan University, Wuhan, Hubei 430072, PR China; College of Chemistry and Molecular Sciences, Wuhan University, Wuhan, Hubei 430072, PR China; State Key Laboratory of Metabolism and Regulation in Complex Organisms, Taikang Center for Life and Medical Sciences, Wuhan University, Wuhan, Hubei 430071, PR China; Department of Hematology, Zhongnan Hospital, Wuhan University, Wuhan, Hubei 430071, PR China

## Abstract

Adenosine base editors (ABEs) facilitate A**·**T to G**·**C base pair conversion with significant therapeutic potential for correcting pathogenic point mutations in human genetic diseases, such as sickle cell anemia and β-thalassemia. Unlike CRISPR–Cas9 systems that induce double-strand breaks, ABEs operate through precise deamination, avoiding chromosomal instability. However, the off-target editing effects of ABEs remain inadequately characterized. In this study, we present a biochemical method Selict-seq, designed to evaluate genome-wide off-target editing by ABEs. Selict-seq specifically captures deoxyinosine-containing single-stranded DNA and precisely identifies deoxyadenosine-to-deoxyinosine (dA-to-dI) mutation sites, elucidating the off-target effects induced by ABEs. Through investigations involving three single-guide RNAs, we identified numerous unexpected off-target edits both within and outside the protospacer regions. Notably, ABE8e(V106W) exhibited distinct off-target characteristics, including high editing rates (>10%) at previously unreported sites (e.g. RNF2 and EMX1) and out-of-protospacer mutations. These findings significantly advance our understanding of the off-target landscape associated with ABEs. In summary, our approach enables an unbiased analysis of the ABE editome and provides a widely applicable tool for specificity evaluation of various emerging genome editing technologies that produce intermediate products as deoxyinosine.

## Graphical Abstract

**Graphical Abstract:**
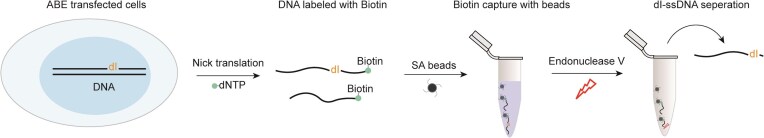

## Introduction

CRISPR-mediated genome editing tools have emerged as potential therapeutic approaches [1–3]. Multiple methods—for instance, GUIDE-seq [4], Digenome-seq [5], and DISCOVER-seq [6]—have reported off-target effects of Cas9. The development of these tools has greatly enhanced our knowledge of genome editing and has provided valuable guidance for developing more reliable editing techniques. Base editing is a newer genome editing approach to directly install point mutations, created by tethering Cas9 nickase (nCas9) to base deaminase enzyme [7]. Although base editors are believed to be safer than the CRISPR–Cas system, as they do not generate double-stranded breaks [8], numerous studies have found that cytosine base editors (CBEs) exhibit evident off-target effects [9–12]. ABE7.10 was reported to induce less off-target sites in rice and mouse embryos [13, 14]; recent studies demonstrate that engineered adenosine base editor (ABE) complexes will induce different mutation landscapes [15, 16]. To identify DNA off-target sites, EndoV-seq investigated *in vitro* genome editing of ABE by selectively cleaving deoxyinosine with Endonuclease V (EndoV) and evaluated ABE specificity through whole-genome sequencing (WGS). However, this method has been shown to suffer from high false-positive rates and low sensitivity.

Before therapeutic applications, a thorough understanding of the genome-wide off-target effects caused by ABE is necessary [17]. As novel modalities of ABEs keep emerging [18], a systematic tool for the direct detection of ABE specificity is lacking. In response to this need, we develop Selict-seq (sequencing of selectively enriched dI-containing ssDNA) to identify genome-wide off-target sites of ABE. Selict-seq achieves selective enrichment of dI-containing single-stranded DNA (ssDNA) and substantially enhances the detection of A-to-I editing sites in cellular context, broadening the existing knowledge about ABE off-target effects.

## Materials and methods

### Cell culture

HEK293T (ATCC, CRL-11268) and MCF7 (ATCC, HTB-22) cells were separately maintained in Dulbecco’s modified Eagle medium (DMEM) and Minimum Essential Medium (MEM) supplemented with 10% Fetal Bovine Serum (FBS) and 1% penicillin/streptomycin at 37°C under 5% CO_2_.

### Plasmid construction and sgRNA cloning

The ABE8e (TadA-8e V106W) expression plasmid (Addgene, #138495) and ABE7.10 expression plasmid (Addgene, #102919) were purchased and used for transfection. To construct single-guide RNA (sgRNA) expression cassettes, target-specific sgRNA sequences (HEK293_site_2, EMX1, RNF2, VEGFA3, and HBG) were cloned into an expression vector under the control of a U6 promoter using the Golden Gate method.

### Transfections

For transfection, 6 × 10^5^ HEK293T cells and 2.3 × 10^5^ MCF7 cells were seeded into six-well culture plates for 16 h growth. HEK293T and MCF7 cells were transfected with 3.75 μg of base editor and 1.25 μg of sgRNA plasmids per well using lipo3000 following the manufacturer’s protocol. Cells were then collected after 72 h of transfection. Genomic DNA was extracted using the FastPure Blood/Cell/Tissue/Bacteria DNA Isolation Mini Kit (Vazyme, DC112-02) and stored at −80°C. The editing efficiency at the on-target sites was assessed by Sanger sequencing.

### Model sequences for estimation of enrichment

Single-strand oligonucleotides were purchased from Sangon Biotech, Inc. To label the 3′ end of DNA with biotin, single-strand oligonucleotides containing dI or dA were annealed to form double-stranded DNA with sticky ends, which were subsequently treated with the Klenow enzyme (Thermo Fisher Scientific, EP0052) to incorporate biotin-dUTP (Trilink, N-5001). After purification with DNA Clean & Concentrator (Zymo, D4004), biotinylated oligonucleotides were denaturized and incubated with streptavidin C1 beads (Invitrogen, 65002) for 1 h. The beads were washed three times with 500 μl B&W buffer (5 mM Tris–HCl, pH 7.5, 0.5 mM EDTA, 1 M NaCl, 0.05% Tween 20) and treated with 100 mM NaOH for 10 min. Then, the beads were washed once more with 100 mM NaOH, followed by three additional washes with B&W buffer. Subsequent enzyme cleavage was performed in a 100 μl reaction mixture containing 10 μl of 10× NEB Buffer 4, 1 μl of Tween 20, and 1 μl of EndoV (NEB, M0305S). The supernatant was collected and purified using DNA Clean & Concentrator. dI and dA DNAs were quantified using Hieff qPCR SYBR GREEN Master Mix (Yeasen, 11201ES03) on a real-time polymerase chain reaction (PCR) system.

### Selict-seq

Before nick translation, 5 μg of DNA was treated with 3 μl USER enzyme (NEB, M5505S) for 30 min at 37°C to remove dU and AP sites, as EndoV exhibits slight activity against both dU and AP. DNase I (NEB, M0303S) was diluted 100-fold, and a total mixture containing 2 μl diluted DNase I, 5 μl DNA polymerase I (NEB, M0209L), 20 μM biotin-14-dATP (Invitrogen, 19524016), 20 μM biotin-16-dUTP, 20 μM biotin-16-dGTP (Trilink, N-5010), and biotin-16-AA-2′-dCTP (Trilink, N-5002) was prepared in nick translation reaction buffer (0.5 M Tris–HCl, pH 7.5, 0.1 M MgCl_2_, 10 mM Dithiothreitol (DTT), 0.5 mg/ml Bovine Serum Albumin (BSA)). After mixing and centrifuging the tube, treated genomic DNA was added and incubated for 2 h at 15°C. The reaction was stopped by adding 2 μl of 0.5 M EDTA and the fragmented DNA was purified by DNA Clean & Concentrator.

To capture the labeled fragments, streptavidin C1 beads (100 μl, Invitrogen) were pre-washed three times with 1× B&W buffer (5 mM Tris, pH 7.5, 0.5 mM EDTA, 1 M NaCl, 0.05% Tween 20) and then resuspended in 50 μl of 2× B&W buffer (10 mM Tris, pH 7.5, 1 mM EDTA, 2 M NaCl, 0.1% Tween 20). The beads were incubated with the resuspended samples at room temperature for 1 h. The beads were washed three times with 1× B&W buffer (5 mM Tris–HCl, pH 7.5, 0.5 mM EDTA, 1 M NaCl, 0.05% Tween 20), followed by treatment of 100 mM NaOH for 10 min at room temperature. Beads were then washed another three times with 1× B&W buffer and incubated with 5 μl EndoV and 1% Tween 20 in 1× NEB Buffer 4 at 20°C for 30 min. Beads were then washed once with 50 μl of 100 mM EDTA (effectively chelates residual Mg^2+^ from reaction buffer) and once with 50 μl of 100 mM NaOH (fully releasing ssDNA from streptavidin–biotin complexes). The supernatants were combined and then purified with Oligo Clean & Concentrator and dissolved in 10 μl of nuclease-free water.

### ssDNA library construction

Library construction was performed according to the ssDNA library construction protocol with some modifications [19]. Adapters and primers (Supplementary Table S1) were purchased from Takara Bio Inc. Two microliters of 100 μM Ad1-F and 2 μl of 200 μM Ad1-R were annealed in 1× T4 RNA ligation buffer in a thermoscycler. Similarly, hybridize 20 μl Ad2-F and 20 μl Ad2-R in 50 mM NaCl. Afterward, eluted DNA was incubated in a 100 μl mixture [1 μl annealed Ad1-F/Ad1-R (10 μM/20 μM), 20% PEG-8000, 0.5 mM ATP, 0.375 U/μl T4 DNA ligase high conc. (NEB, M0437M) in 1× T4 RNA ligation buffer] at 37°C for 1 h and at 95°C for 2 min.

The ligation products were immobilized on streptavidin C1 beads according to the manufacturer’s instructions. Free adapters were removed by washing once with 1× B&W buffer, once with 100 μl stringency wash buffer (0.1% SDS, 0.1× SSC buffer) at 45°C for 3 min, and once with 1× B&W buffer. To synthesize the second strand, the beads were resuspended with 48 μl mixture containing 2 μM primer, 200 μM each dNTP, and 0.05% Tween 20 in 1× Klenow reaction buffer. After 2 min at 65°C, 2 μl Klenow fragment was added and the beads were incubated for 2 min at 25°C and for 25 min at 35°C with gentle shaking. After a second bead wash, the beads were resuspended with 100 μl mixture containing 2 μM each Ad2-F/Ad2-R, 5% PEG-4000, 0.05% Tween 20, and 0.1 U/μl T4 DNA ligase in 1× T4 DNA ligase buffer. After incubating for 1 h at 22°C in a thermomixer (800 rpm), another bead wash was performed with B&W buffer, stringency wash buffer and B&W buffer as described earlier. The DNA was eluted from C1 beads using TET buffer (10mM Tris–HCl pH 8.0, 1 mM EDTA, 0.5% Tween 20) after heating at 95°C for 1 min. Eluted DNA was finally subjected to PCR amplification. Library PCR amplification was performed using the NEB primers for 13 cycles. The products were purified by 0.9× VANTS DNA clean beads and then used for sequencing. Sequencing was performed using an Illumina NovaSeq platform (Azenta Life Sciences) with 150-bp paired-end reads.

### Targeted amplicon sequencing

Primers were designed to amplify the targeting sites, and paired Illumina adapter sequences were added to the primers. Additionally, a 5-nt UMI was added into forward primer and reverse primer, respectively, to lower the detection limit (Supplementary Table S1). The first round of PCR amplification was performed with Q5 Hot Start High-Fidelity 2× Master Mix (NEB, M0494L) using 10 ng of genomic DNA as an input template. After 15 cycles of amplification, the PCR products were purified with 1.5× VANTS DNA clean beads (Vazyme, N411-01) and eluted with TE buffer. Purified DNA samples were then subjected to the second round of amplification for another 15 cycles and assigned with different indexes followed by a purification with 0.8× VANTS DNA clean beads. The libraries were pooled for high-throughput sequencing by Illumina NovaSeq.

### Selict-seq mapping

Illumina sequencing adapters were removed by cutadapt software (v.1.18). Working command and key parameters were as follows: cutadapt --times 1 -e 0.1 -O 3 --quality-cutoff 25 -m 30. After the adapter removal, FASTQ files were mapped to the reference genome (hg38) using bowtie2 with parameter “--end-to-end.” Only the proper pair and primary alignments persisted for the downstream pipelines. The BAM files were sorted by reference coordinate with samtools sort command (v.1.9). Duplications were removed from the sorted BAM files by Picard MarkDuplicates (v.2.0.1).

### Identification of dA-to-dI sites

A custom script was used to parse the pileup format into a tabular format summarizing the mutation at each position. We focused on processing the R1 reads. Reads on the positive and negative strands of the reference genome were analyzed separately. To evaluate the distribution of the second base initiated by truncated reads, a series of detection-based thresholds were established: (i) minimum mapping quality of five; (ii) minimum read coverage per candidate dI site to be five; (iii) the mutation number of candidate dI sites was required of at least three, with a proportion not less than 20%; (iv) sites that appear in the background sample are no more than one; (v) candidate dI sites must be present in both replicated samples; (vi) to reduce sequencing and alignment errors, we excluded three consecutive mutations of different types with a mutation rate of >20%; and (vii) known single nucleotide polymorphism sites (dbSNP 151) were removed. Finally, A-to-G mutations in the positive strand and T-to-C mutations in the negative strand, specifically at the second base of the truncated reads, were identified as candidates for dA-to-dI editing.

### Identification of Cas9-dependent off-target sites

For dA-to-dI editing sites, we initially ranked the off-target sites according to the Selict-seq score [5, 20]. We then sorted the scores for each site, selecting those with scores ≥0.6 and an edited ratio ≥0.2, while the mutation read count in sgRNA(−) samples was no larger than one.

To identify an sgRNA binding site for dA-to-dI editing sites, we extracted sequences spanning 100 nucleotides around the mutation sites from the reference genome hg38 and aligned them with the on-target sequence using a modified semiglobal alignment algorithm [12]. Using the semiglobal alignment parameters set as match +5, mismatch −4, gap open −24, and gap extension −8, we find the most suitable binding sites for each mutation site. To define high-confidence sgRNA binding sites, we retained those containing editing sites and exhibiting an alignment penalty score <70.

### Identification of the Cas9-independent off-target sites

We identified Cas-independent off-target sites by comparing the Selict-seq signals between sgRNA(−) sample and untreated cell samples. First, the Cas9-independent off-target sites required the mutation read count in sgRNA(−) sample to be no fewer than three and the mutation site did not exist in untreated cell samples [the mutation sites observed in both sgRNA(−) and untreated samples were classified as endogenous dI sites (not induced by ABE)]. Subsequently, Cas9-independent sequence data were extracted by Cas9-independent sites using Bedtools getfasta (v.2.31.3), and the motif result was constructed by Meme software (v.5.5.5).

### Effect factor analysis with ridge regression

Ridge linear regression analysis of factors influencing editing outcomes. To find the effective factors of out-of-protospacer edits and target-strand edits, we performed a ridge regression analysis by ridge package (v.3.3) in the R environment (v.4.4.2). All identified off-target sites with ABE8e in the HEK293T cell line were combined for this analysis. For out-of-protospacer edit analysis, the dependent variable was defined as a binary value (zero or one), indicating whether out-of-protospacer edits occurred for each specific off-target site. Then, we selected first 8-bp mismatch count, first 5-bp mismatch count, first 8-bp gap count, first 5-bp gap count, alignment mismatch count, alignment gap count, seed region mismatch count, seed region gap count, and protospacer adjacent motif (PAM) type as the independent factors. Next, we fitted a linear ridge regression model between the dependent variable and the selected independent variables. Factors with a *P*-value lower than .01 were considered significant contributors to out-of-protospacer edits. The steps for conducting the effective factor analysis for target-strand edits followed the same procedure as described for out-of-protospacer edits.

### Targeted amplicon sequencing data analysis

For targeted amplicon sequencing data, we first removed Illumina sequencing adapters in raw FASTQ data with fastp software (v.0.23.2), and then combined the single-end data to paired-end data with seqtk software (v.1.4). For reads in the same UMI group, we corrected sequencing errors and removed PCR duplications to improve the detection limit by seqkit software (v2.6.1), and then the UMI sequences were removed with cutadapt software (v.4.1). Cleaned reads were mapped to the reference index by BWA MEM (v.0.7.17) with default parameters. Next, we generated bamcount files from mapped BAM files using the bam-readcount software (v.1.9). Editing rates were calculated as (edited reads)/(total qualified reads) × 100% at each position, with background subtraction using matched untreated controls. Only sites with ≥500× coverage in both test and control samples were included for analysis.

## Results

### Assessing genome-wide ABE editing events by Selict-seq

**Figure 1.**
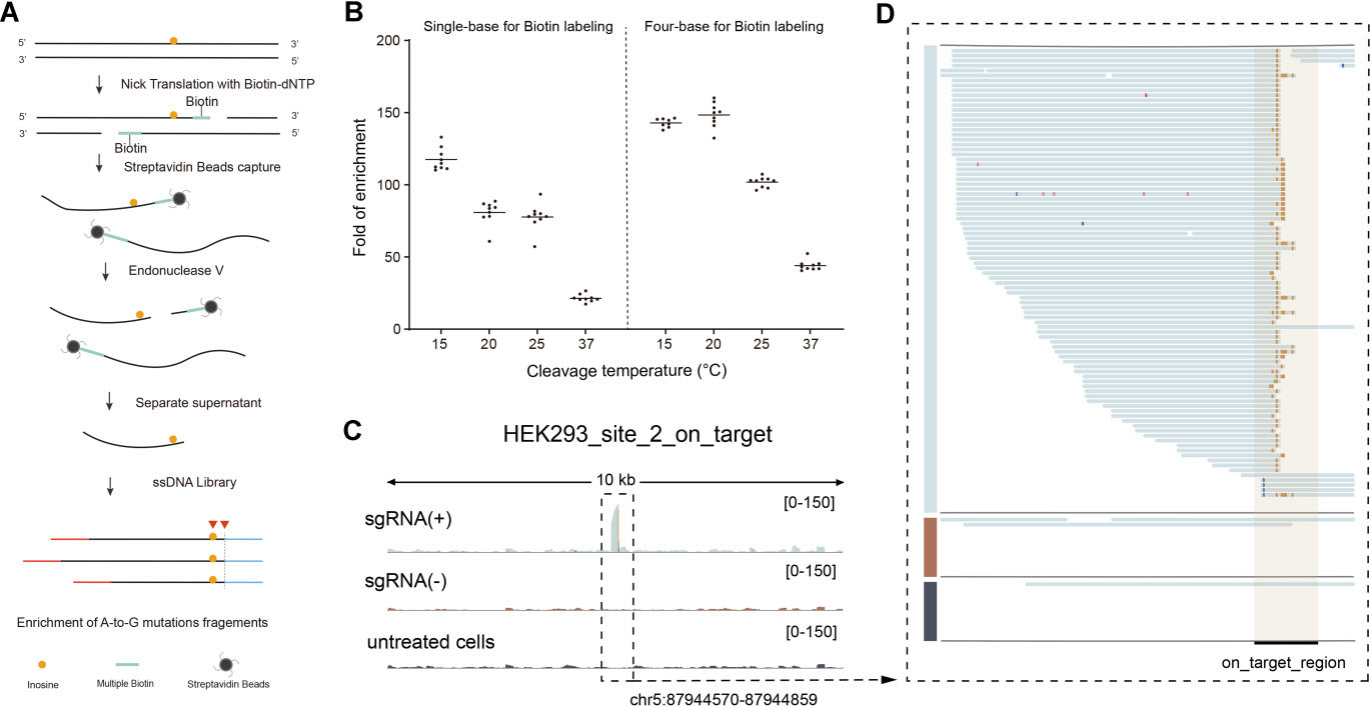
Assessing genome-wide ABE editing events by Selict-seq. (**A**) Schematic overview of the Selict-seq method. (**B**) Enrichment fold of dI-containing model sequences by Selict-seq. Values are calculated by the 2^−ΔΔCt^ method with normalization through a control sequence that only consists of canonical bases. Each dot represents an independent sample; horizontal lines represent the mean values. (**C**) The enriched peaks and Selict-seq signals at the on-target site for HEK293T_site_2 sgRNA are shown in Integrative Genomics Viewer (IGV), covering a 10-kb region around the sgRNA on hg38 genome with a maximum coverage track of 150. The region highlighted by the dashed lines is detailed in panel (D). (**D**) The IGV displays the alignment of sequencing reads at the HEK293T_site_2 sgRNA target region. Blocks on the nontarget strand indicate A-to-G mutations, while blocks on the target strand represent T-to-C mutations. The sgRNA(+) data are presented at the top, the sgRNA(−) data in the middle, and the untreated cells data at the bottom. The sgRNA binding region is outlined (gray rectangle).

ABEs rely on the adenosine deaminase (*Escherichia coli* TadA) to convert dA to dI on the PAM-containing strand, and finally result in dA-to-dG transitions [21]. EndoV can specifically recognize dI and cleave at the second base on the 3′ side [22]. We hypothesized that DNA fragments are labeled with biotin, allowing them to be captured by magnetic beads. Then, dI-containing ssDNA can be separated from the genomic DNA by EndoV, thereby achieving enrichment (Fig. 1A). Because EndoV exhibits activity toward several DNA structures that may form during ssDNA self-folding [23], we used model sequences to evaluate the system and optimized the condition. The dI-containing model sequences and control sequences were labeled with biotin-dUTP using Klenow (exo-)polymerase and subsequently treated with EndoV for cleavage (Supplementary Fig. S1). We found that reducing reaction temperature could enhance EndoV selectivity for dI-containing ssDNA. Under the optimized conditions, dI-containing DNA could be enriched ∼150-fold compared to the dA-containing DNA (Fig. 1B). Moreover, we observed an enhanced enrichment fold at model sequences with four-base overhangs, suggesting that multiple biotin labels improve the enrichment efficiency (Fig. 1B).

We labeled genomic DNA by nick translation [24], which can incorporate multiple nucleic acid modifications at the 3′ ends of the DNA fragments. To ensure the conversion of DNA into appropriately sized ssDNA fragments, we optimized the nick translation conditions. As shown by alkaline agarose gel electrophoresis, DNA was sufficiently fragmented under the condition of 100-fold diluted DNase I (Supplementary Fig. S2 and see the “Materials and methods” section). Genomic DNA was first subjected to USER enzyme to eliminate interference from abasic sites (AP) and deoxyuridine (dU). Then, we labeled genomic DNA with biotin-dNTPs at 3′ end (Fig. 1A and Supplementary Fig. S3A). Multiple biotin labels allow genomic DNA immobilized to the magnetic beads. During this process, unbiotinylated ssDNA is rigorously washed away (Supplementary Fig. S3A). EndoV selectively cleaves dI-containing ssDNA from the beads for subsequent collection and purification. Then, we construct a library of truncated ssDNA using the ssDNA library protocol (Supplementary Fig. S3B) [19].

We then applied Selict-seq to evaluate the off-target effects of ABE8e(V106W) [25] in HEK293T cells for several frequently used sgRNAs—HEK293_site_2, EMX1, and RNF2 [noted as “sgRNA(+)”]. A negative control without sgRNA [sgRNA(−), ABE8e(V106W) transfected without sgRNA] and a background control (untreated cells without ABE or sgRNA) were included to eliminate endogenous dI signals and technical noise. As expected, we observed peaks at the on-target sites in HEK293_site_2 sgRNA(+) samples (Fig. 1C). Additionally, we found characteristic truncated dA-to-dG mutation patterns (Selict-seq signal) at the on-target site, where A-to-G mutations predominantly occur at the second nucleotide position of the reads, showing a significant enrichment signal (Fig. 1D). Then, we searched the whole genome for mutation sites with characteristic Selict-seq signal and compared the sgRNA(+) samples with the sgRNA(−) samples. We found that all three sgRNA(+) samples for these dA-to-dG mutations dropped to background level when sgRNAs were omitted (Supplementary Fig. S4), indicating that these mutation sites are Cas9-dependent off-target sites.

### Selict-seq sensitively profiles genome-wide off-target sites of ABE

We were subsequently inspired to generate a pipeline to identify off-target editing sites. First, we defined a Selict-seq score [5, 20], computed as the normalized number of reads containing truncated mutation signals at a given site. The Selict-seq score was then used to evaluate the reliability of the dA-to-dI editing sites (Supplementary Fig. S5 and see the “Materials and methods” section). Then, we searched candidate binding regions for each sgRNA around the identified dA-to-dI sites, and most dA-to-dI sites were found within the editing window with high confidence (Supplementary Table S2 and see the “Materials and methods” section).

**Figure 2.**
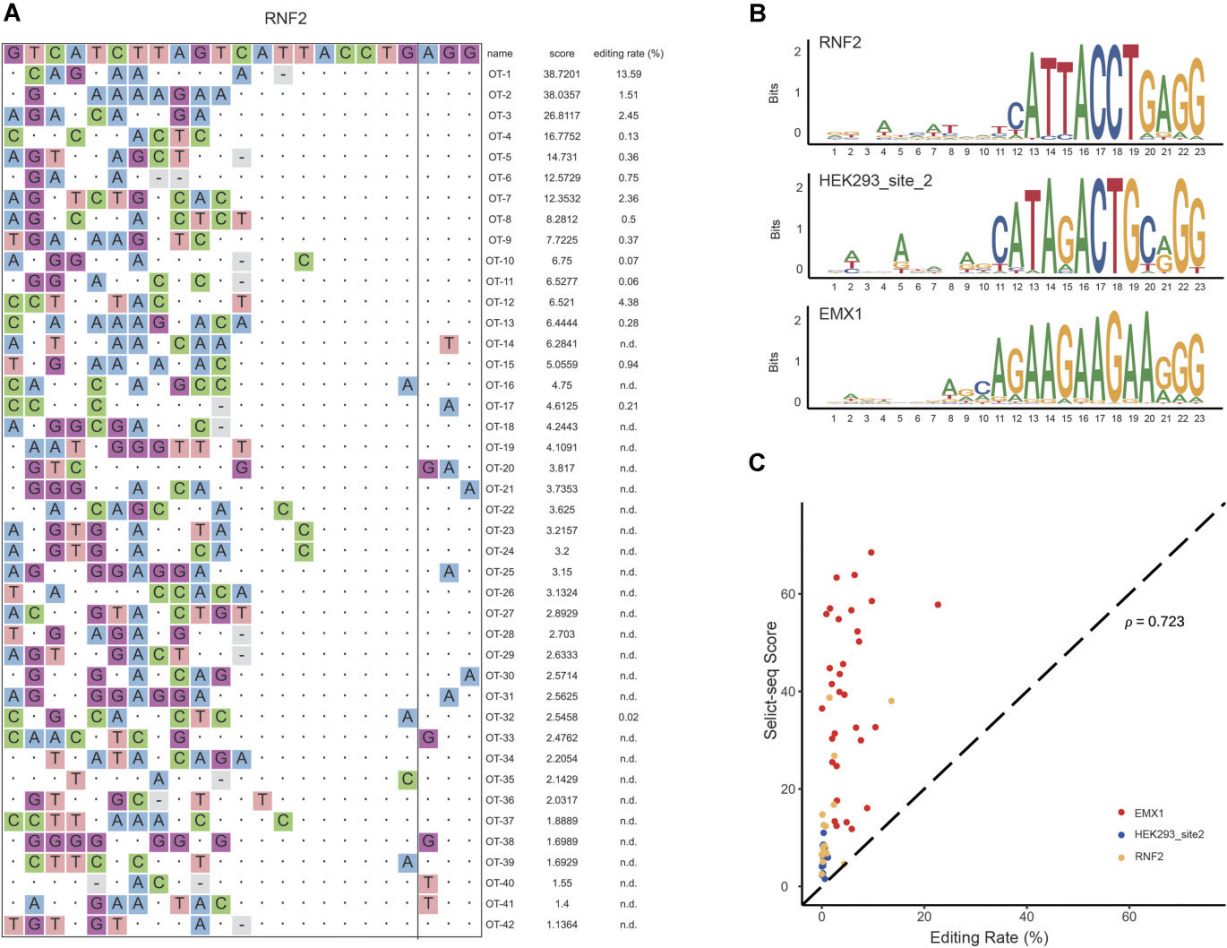
Selict-seq sensitively profiles genome-wide off-target sites of ABE8e. (**A**) Off-target sequences identified by Selict-seq for RNF2 sgRNA. The on-target sequence is shown at the top. Mismatched bases are annotated below each sequence. Putative sgRNA bulges are shown in gray. Selict-seq scores and editing rates are shown to the right of each site; n.d., not determined because of difficulties in PCR amplification. (**B**) Sequence logos of sgRNA targeting regions for RNF2, HEK293_site_2, and EMX1 sgRNAs. (**C**) Selict-seq scores and editing rates obtained by targeted amplicon sequencing are correlated (Spearman correlation).

For HEK293_site_2 and EMX1 sgRNAs, we detected 19 and 726 off-target sites, respectively, with dA-to-dI editing events occurring within their respective protospacer regions (Supplementary Table S2). Targeted amplicon sequencing [26] was conducted on all HEK293_site_2 sites and a subset of EMX1 sites, successfully amplifying 12 and 34 off-target sites for HEK293_site_2 and EMX1, respectively (Supplementary Fig. S6). Among the sites validated for HEK293_site_2 sgRNA, 11 out of 12 (91.7% true positive rate, TPR) exhibited editing rates above the background level (>0.2%), with 1 site exceeding 1% (Supplementary Fig. S6A). For the EMX1 sgRNA, 33 out of 34 amplifiable sites (97.1% TPR) showed detectable editing, with 17 sites exceeding 5% and 1 reaching 24.79% (Supplementary Fig. S6B–D). For the RNF2 sgRNA, which previous studies using GUIDE-seq [4] and Digenome-seq [6] reported as having no detectable off-target effects in CRISPR–Cas9, we detected 42 off-target editing sites (Fig. 2A). Using targeted amplicon sequencing, we successfully amplified 16 of these sites, with 12 out of 16 (75% TPR) showing editing rates above background levels. Notably, the most severe editing rate exceeded 10%, with five sites showing editing rates above 1% (Fig. 2A). Editing efficiencies of particular off-target sites for EMX1 and RNF2 are actually higher than their corresponding on-target sites (Fig. 2A and Supplementary Figs S6B and S7), probably caused by accessibility of the target DNA [9, 27].

We obtained a sequence logo for RNF2, HEK293_site 2, and EMX1 sgRNA via WebLogo using DNA sequences of the sgRNA binding sites (Fig. 2B), demonstrating lower sensitivity of ABE8e(V106W) to sequence mismatches in PAM-distal regions compared with canonical Cas9 nuclease. Also, we observed a strong correlation between Selict-seq scores and off-target editing ratio (Fig. 2C), showing the potential of Selict-seq to estimate the *in vivo* editing levels of ABE.

### Comparison of Selict-seq with existing methods

**Figure 3.**
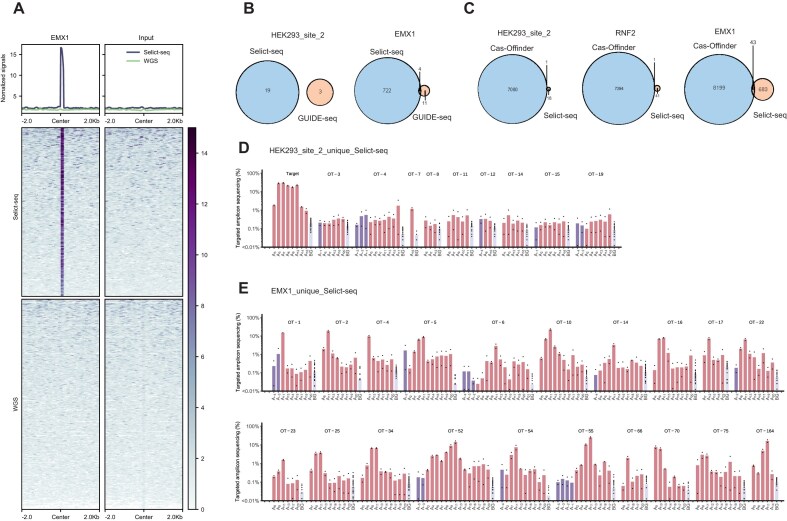
Comparison of Selict-seq with existing methods. (**A**) Heatmaps of normalized signals in Selict-seq and WGS at an identified off-target site by Selict-seq within a 4-kb window. The panels from left to right, respectively, show signals in sgRNA(+) samples and sgRNA(−) samples. (**B**) Venn diagrams that compare off-target sites identified by GUIDE-seq and Selict-seq for HEK293T_site_2 and EMX1 sgRNAs. (**C**) Venn diagrams that compare off-target sites identified by Cas-OFFinder and Selict-seq for HEK293T_site_2, RNF2, and EMX1. Editing rates of unique off-target sites identified by Selict-seq for (**D**) HEK293T_site_2 and (**E**) EMX1. Adenosine positions are categorized as: outside the protospacer, within the protospacer, or background (adenosines from untreated cell controls).

We subsequently compared Selict-seq signals at off-target sites with signals obtained from WGS (Fig. 3A). Our analysis revealed significantly stronger signals in Selict-seq compared to background signals or WGS at off-target site (Fig. 3A and Supplementary Fig. S8).

We also compared our results with GUIDE-seq [4], which verified the off-target effects for HEK293_site_2 and EMX1 sgRNA. For HEK293_site_2, we found no shared off-target sites with GUIDE-seq; for EMX1, we found only four off-target sites that are also reported by GUIDE-seq (Fig. 3B). Attempts to amplify GUIDE-seq unique sites for HEK293_site_2 failed, and we found these sites with no Selict-seq signal (Supplementary Fig. S9). Unique off-target sites by Selict-seq show editing rates from 0.21% to 1.18% (Fig. 3D). For EMX1, targeted amplicon sequencing confirmed high editing rates at 20 Selict-seq-identified sites, with multiple editing sites in the sgRNA binding region (Fig. 3E). We also validated the four shared off-target sites for EMX1, which had editing rates between 0.7% and 10% (Supplementary Fig. S10A). For unique off-target sites identified by GUIDE-seq, targeted sequencing showed that 10 out of 11 sites had editing rates below 0.2%, suggesting that no real editing events occurred (Supplementary Fig. S10B). Besides, we checked the GUIDE-seq OT-2 site using Selict-seq data and found that the original data did show editing signals (Supplementary Fig. S10C). However, these signals were filtered out because they did not pass the threshold in one of the replicates and consequently failed our bioinformatic criteria.

Furthermore, we compared Selict-seq and EndoV-seq [28] using an identical base editor (ABE7.10) and four sgRNAs (EMX1, HBG, VEGFA3, and RNF2). Selict-seq detected 147, 24, 11, and 2 editing sites, respectively (Supplementary Fig. S11A), with almost no overlap with those identified by EndoV-seq. Additionally, Selicit-seq revealed that the sensitivity of ABE7.10 to sequence mismatches in PAM-distal regions differs from that observed with EndoV-seq (Supplementary Fig. S11B). We speculate that the significant discrepancy between the two methods may stem from differences between *in vivo* editing detection (Selict-seq) and *in vitro* editing detection (EndoV-seq) assays, as reported in previous studies [20].

We then analyzed off-target effects by ABE7.10 and ABE8e and found that ABE7.10 identified fewer off-target sites across different sgRNAs compared to ABE8e (Supplementary Fig. S12), consistent with previously reported ABE activity [25]. Notably, a substantial overlap in detected sites was observed between different ABEs. Specifically, for the EMX1, RNF2, VEGFA3, and HBG sgRNAs, there was a significant overlap, with 146 out of 147 sites, 2 out of 2 sites, 9 out of 11 sites, and 24 out of 24 sites overlapping, respectively (Supplementary Fig. S12). This underscores the robustness of our detection method across different ABE versions and highlights the consistency and reliability of our approach.

Cas-OFFinder [29], a widely utilized off-target prediction tool, was employed to compare results with Selict-seq findings. Despite Cas-OFFinder predicting thousands of off-target sites for different sgRNAs, only a small subset of the detected sites overlapped with these predictions (Fig. 3C), possibly due to the limitations of Cas-OFFinder in predicting off-target sites with numerous mismatches or larger DNA/RNA bulges. This difference emphasizes how various factors affect off-target editing and reveals the ongoing difficulties in accurately forecasting off-target events. Overall, these comparisons highlight the high specificity and sensitivity of Selict-seq in identifying ABE off-target effects.

To evaluate Selict-seq’s versatility across cellular contexts, we transfected ABE8e and sgRNAs (EMX1, RNF2) in MCF7 cells. Selict-seq identified 338 off-target sites for EMX1 and 10 for RNF2, of which 20 sites were unique to MCF7 compared to HEK293T (Supplementary Fig. S13A). These sites exhibited similar sequence motifs (Supplementary Fig. S13B), suggesting that the observed differences are attributable to different cellular environments rather than sequence variations.

### Features of ABE8e-induced off-target sites

**Figure 4.**
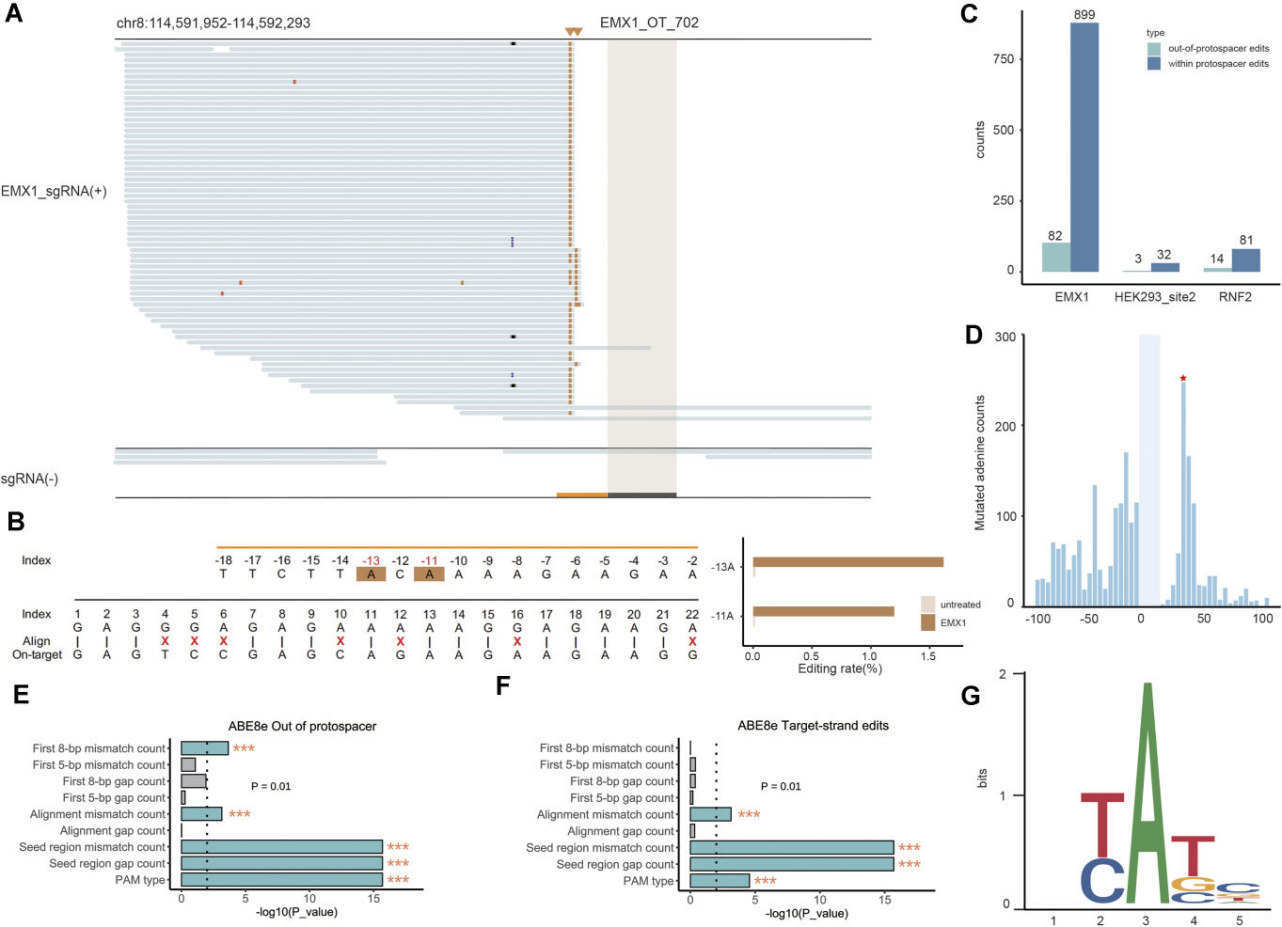
Features of ABE-induced off-target sites. (**A**) An example of ABE edits outside the protospacer. The editing window is shaded. Edits outside the protospacer are marked with inverted triangles. (**B**) Sequence of genomic region shown in panel (A). Editing rates of the out-of-protospacer edits are shown to the right. (**C**) Counts of editing events outside the protospacer as well as edits within the editing window for all aligned sgRNA binding regions. (**D**) Distribution of out-of-protospacer editing and edits within the protospacer. The asterisk indicates the most frequent out-of-protospacer edits. Effects of mismatch/gap count and position as well as PAM type on (**E**) out-of-protospacer editing and (**F**) target-strand editing, estimated by ridge linear regression analysis. *P*-values were calculated by two-sided Student’s *t*-test. (**G** ) The sequencing context of sgRNA-independent off-target edits. The flanking sequences (2  bp on either side, fixing the mutated adenosine in each case at position 3; *n* = 58 cases) were extracted from the hg38 reference genome to produce a sequence logo using WebLogo.

In our targeted sequencing analysis for the ABE8e off-target sites, we identified several out-of-protospacer off-target sites exhibiting high editing rates (Fig. 3D and E). In one site, adjacent to the sgRNA binding site, we observed significant Selict-seq signals (Fig. 4A) with an editing rate of 1%–2% confirmed by targeted amplicon sequencing (Fig. 4B). For the three sgRNAs, we identified 82, 3, and 14 out-of-protospacer off-target sites in EMX1, HEK293_site_2, and RNF2, respectively (Fig. 4C). Additionally, we calculated the relative distances of all mutation signals from sgRNA binding regions for three sgRNAs (Fig. 4D). The mutation signals were predominantly concentrated upstream to the protospacer. For the downstream editing sites with highest number of mutated adenosine read counts, we found that most of these sites have noncanonical PAM sequences (not NRG) (Supplementary Fig. S14A and B, and Supplementary Table S2).

We also found both A-to-G and T-to-C mutations in one region (Supplementary Fig. S14C), indicating editing events happened on both the target strand and nontarget strand. Then, we searched Selict-seq signals on the target strand and identified 3, 3, and 8 target-strand edits for EMX1, HEK293_site_2, and RNF2, respectively (Supplementary Table S2). Moreover, we found most of these target-strand edits located outside the protospacer (Supplementary Table S2).

To investigate the mechanism of editing outside the protospacer, we conducted an in-depth analysis of our results. Ridge linear regression analysis demonstrated that counts of mismatches and gaps at the seed region, as well as noncanonical PAM types at the sgRNA binding sites, are highly correlated with editing outside the protospacer (*P* < 2.0 × 10^−16^). Additionally, the first 8-bp mismatch count (*P* = .00022) and alignment mismatch count *(P* = .0007) also contribute to editing outside the protospacer, although their effects are relatively minor (Fig. 4E). For target-strand edits, off-target edits are influenced by alignment mismatch count (*P* = .00072), PAM type (*P*= .000027), and particularly mismatches and gaps at the seed region (*P* < 2.0 × 10^−16^) (Fig. 4F). We speculated that these mismatches or gaps at the PAM-proximal regions lead to ssDNA bubbles, serving as potential TadA substrates.

Then, we conducted an analysis for dA-to-dG mutation sites present in the sgRNA(−) controls, which are considered to be sgRNA-independent off-target sites. The Selict-seq scores for gRNA-independent off-target sites are lower compared to Cas9-dependent off-target sites, indicating a decrease in editing activity (Supplementary Fig. S15A). Further annotation of the distribution of these sites revealed that both Cas9-dependent and Cas9-independent off-target edits are more enriched in gene regions, such as exons, introns, and promoters, compared to background mutations (Supplementary Fig. S15B). By expanding the surrounding sequences, we identified the base preferences adjacent to each mutation site. Specifically, the +1 position exhibited a preference for T over C, whereas the −1 position showed a preference hierarchy of T >> G > C (Fig. 4G), highlighting a propensity of ABE8e toward T bases for the −1 and +1 positions. ABE7.10 has been reported to prefer 5′YAY (Y = T/C) sequences [30]. In fact, ABE8e shows noticeable preferential editing in a TA motif [31]. Our results demonstrated a TAT motif for sgRNA-independent off-target edits, showing the potential of Selict-seq to comprehensively assess genome-wide off-target effects for ABE complex.

## Discussion

ABEs can introduce precise A-T to G-C substitutions at targeted genomic sites by enabling the direct conversion of adenosine to inosine. This capability positions ABEs as a powerful therapeutic tool with the potential to correct ∼47% of known pathogenic single nucleotide variations (SNVs) [32, 33]. The rapid development of ABEs highlights their great potential for future clinical research and therapeutic applications [34–36]. Highly efficient and precise correction of single-nucleotide pathogenic mutation is demanded for gene therapy to reach its potential. However, ABE-induced off-target effects, including unintended genomic alterations and potential oncogenic risks, impede their clinical translation and therapeutic reliability. In this study, we present an unbiased approach to characterize the ABE editome by selectively capturing dI-containing ssDNA. Our results identified genome-wide off-target sites induced by ABE and verified the mutation landscapes for various sgRNAs. The identification of off-target sites in different versions of ABE (ABE8e versus ABE7.10) exhibited a significant overlap, demonstrating a strong consistency of Selict-seq. Particularly, we found multiple off-target sites for RNF2 sgRNA (ABE8e), which was reported editing without off-target effect [4, 6]. Compared to methods combining EndoV with Cas9 cleavage activity (e.g. CHANGE-seq [37] and EndoV-seq [28]), Selict-seq requires additional control (sgRNA-negative) to identify Cas9-dependent off-targets. However, Selict-seq directly profiles ABE activity in living cells, providing a physiologically relevant assessment of editing outcomes. Consequently, we recommend a systematic assessment about ABE specifically before any therapeutic application. Our results found that ABE complex induces most editing events within the editing window, but also catalyzes off-target edits outside the editing window on both sgRNA-binding strand and PAM-containing strand. Noncanonical PAM types seem to induce out-of-protospacer edits at a certain distance to the editing window. Also, we found a characteristic motif for sgRNA-independent off-target sites, broadening the knowledge about ABE specificity.

Selict-seq provides a robust approach to directly detect ABE-induced off-target sites. Although ABE has been reported with less off-target mutations compared to CBE [13, 14], our study detected considerable off-target effects induced by ABEs, underscoring the necessity for thorough assessment before clinical applications. Moreover, we anticipate that Selict-seq can be applied to a wide range of gene editing methodologies that introduce dI, such as ACBE [38], and mitochondrial base editors, including TALEDs [39, 40] and mitoBE [41]. Additionally, we propose that combining Selict-seq with assays detecting ssDNA in DNA damage repair, such as Tracking-seq [42], could offer a more comprehensive scan of ABE off-targets. While ABEs are advancing rapidly, significant progress remains for their therapeutic applications. Selict-seq is inherently compatible with various cell types and tissues, providing a sensitive solution for ABE off-target detection and facilitating the integration of ABEs into clinical applications.

## Supplementary Material

gkaf281_Supplemental_Files

## Data Availability

All data sets have been deposited in the Gene Expression Omnibus under accession number GSE278492. Code for the analyses described in this paper is available in the GitHub repository (https://github.com/whu-XiXin/Selict-seq).

## Supplementary data

Supplementary data is available at NAR online.
