# Supplementary material for: Selict-seq profiles genome-wide off-target effects in adenosine base editing
Source: Nucleic Acids Res. 2025 Apr 10;53(7):gkaf281. doi: 10.1093/nar/gkaf281 (PMC11983105; doi:10.1093/nar/gkaf281)
Supplement: gkaf281_Supplemental_Files [file gkaf281_supplemental_files.zip › NAR_SI_revised 2.pdf]

Selict-seq profiles genome-wide off-target effects in adenosine base editing

Kexin Yuan[1,†], Xin Xi[1,†], Shaoqing Han[1, †], Jingyu Han[1], Bin Zhao[1], Qi Wei[1,*], Xiang Zhou[1,2,3,*]

[1] College of Chemistry and Molecular Sciences, Wuhan University, Wuhan, Hubei 430071, PR China
[2] State Key Laboratory of Metabolism and Regulation in Complex Organisms, Taikang Center for Life and Medical Sciences, Wuhan University, Wuhan, Hubei 430072, PR China
[3] Department of Hematology, Zhongnan Hospital, Wuhan University, Wuhan, Hubei 430071, PR China

*To whom correspondence should be addressed. Tel: +86 27 68756663; Fax: +86 27 68756663; Email: xzhou@whu.edu.cn. Correspondence may also be addressed to Qi Wei. Email: qwei@whu.edu.cn.
† These authors contributed equally to this work.

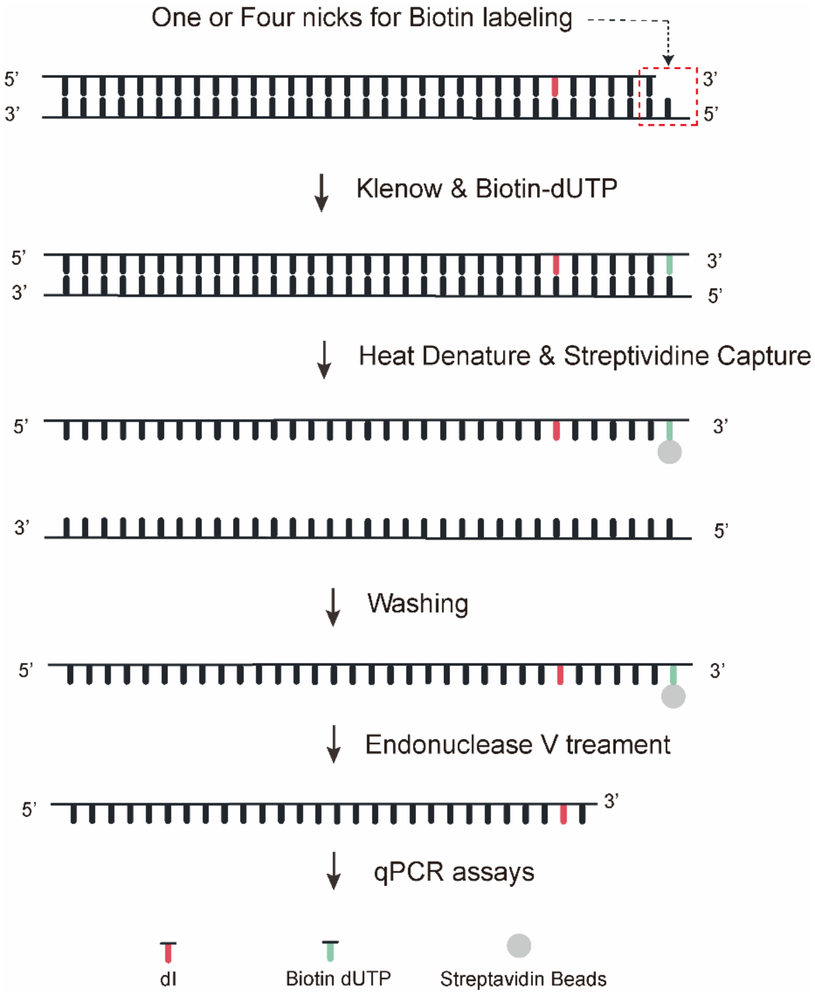

Supplementary Figure S1. Optimization of Selict-seq by quantitive PCR experiments. Schematic procedures to optimize Selict-seq by quantifying the enrichment-fold of dI-containing model sequences by qPCR experiments. Single-stranded model sequences are annealed to form double-stranded DNA with 1-base overhang or 4-base overhang.

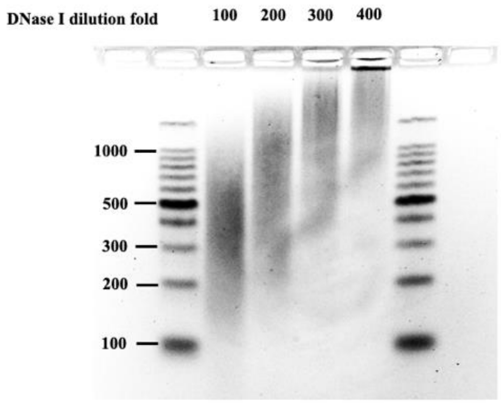

Supplementary Figure S2. Genomic DNA of HEK293T cells was used to test the length of DNA fragments after Nick-translation. The genomic DNA (5 μg) was incubated with dNTP, E.coli DNA polymerase I and diluted DNase I (100-, 200-, 300- and 400-fold) at 20℃ for 2 h. The treated products were purified and resolved by alkaline agarose gel electrophoresis. Molecular weight marker size is in nucleotides.

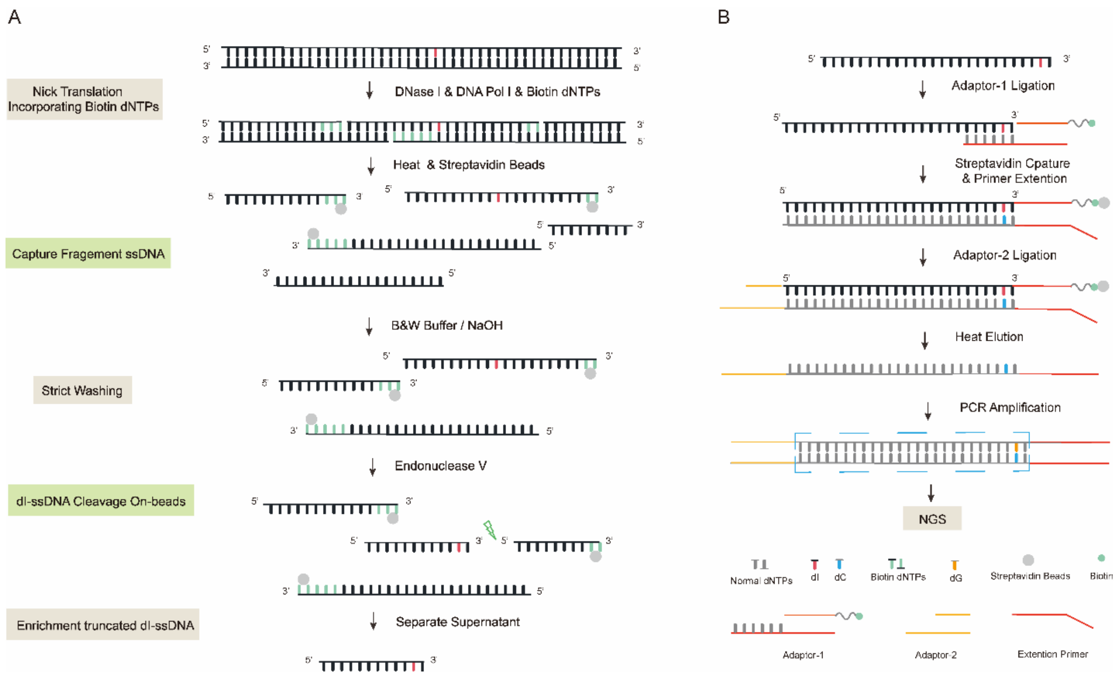

Supplementary Figure S3. Overview of Selict-seq. (A) A schematic procedure of Selict-seq. **(B)** Schematic overview for construction of single-stranded DNA library.

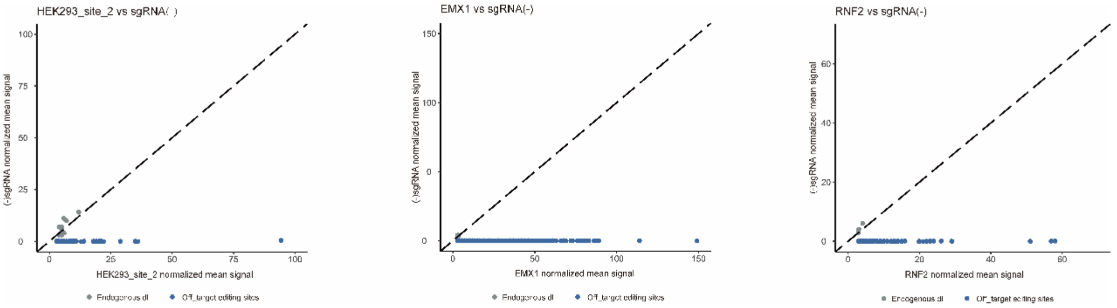

Supplementary Figure S4. The identified dA-to-dG mutations are experimentally proved to be Cas9-dependent. Comparison of Selict-seq signal intensities between sgRNA(+) samples and sgRNA(−) samples among three sgRNAs transfected in HEK293T cell lines. Points below the diagonal indicate loci show reduced signal intensity upon omitting sgRNA, colored in blue. In contrast, points stay at the diagonal indicate endogenous dI, colored in gray.

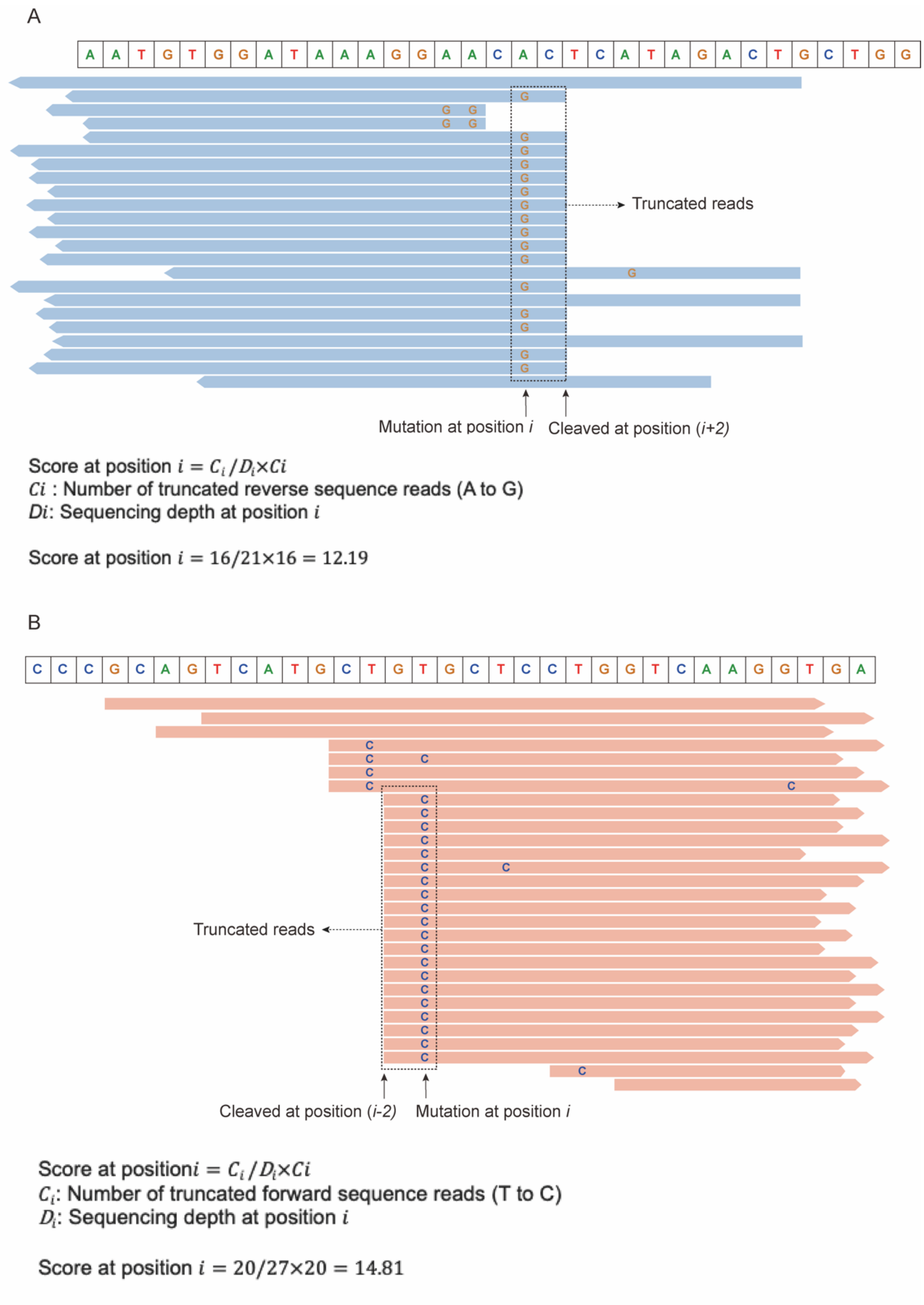

Supplementary Figure S5. DNA cleavage scoring system for Selict-seq analysis of ABE. (A) The number of reverse sequence reads with cleavage signal at a given locus (A-to-G) under normalized sequencing depth is calculated to evaluate the reliability of non-target strand edits. (**B)** The number of forward sequence reads with cleavage signal at a given locus (T-to-C)

under normalized sequencing depth is calculated to evaluate the reliability of target-strand edits.

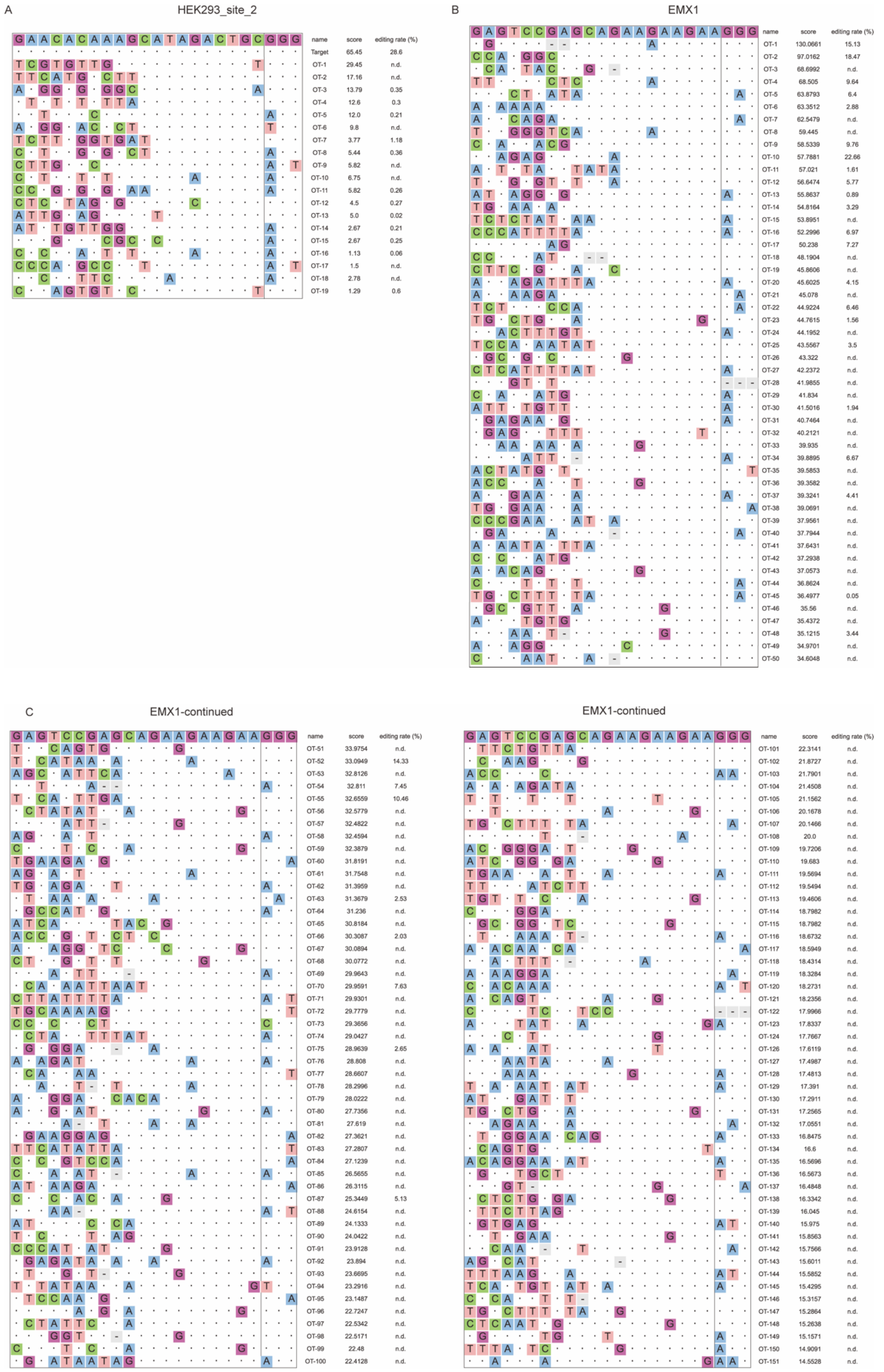

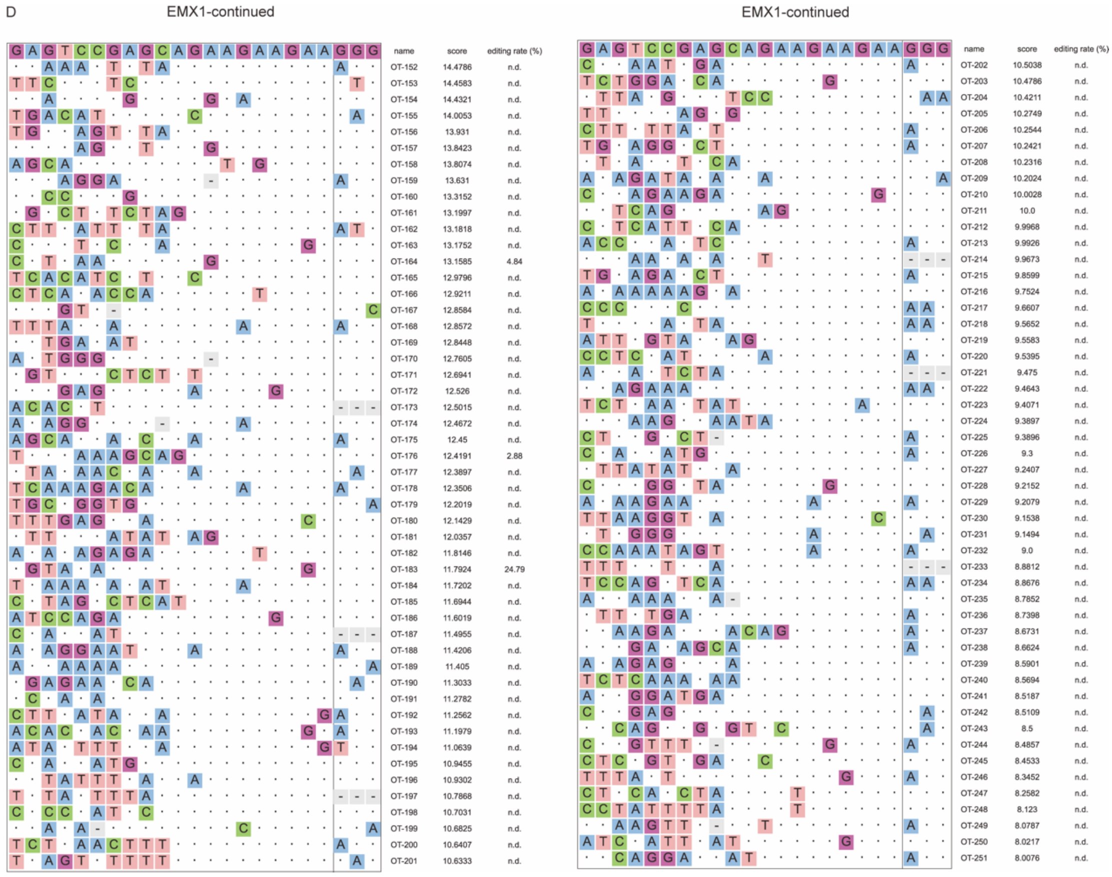

Supplementary Figure S6. Off-target sequences identified by Selict-seq. (A,B,C,D) Off-target sequences for HEK293_site_2 sgRNA **(A)** and EMX1 sgRNA **(B,C,D)**. The on-target sequence is shown at the top of the alignment. The mismatches are highlighted in color. The putative sgRNA bulges are shown in gray. Corresponding Selict-seq scores and editing ratios evaluated by targeted amplicon sequencing are listed to the right of each site. n.d. means not detected.

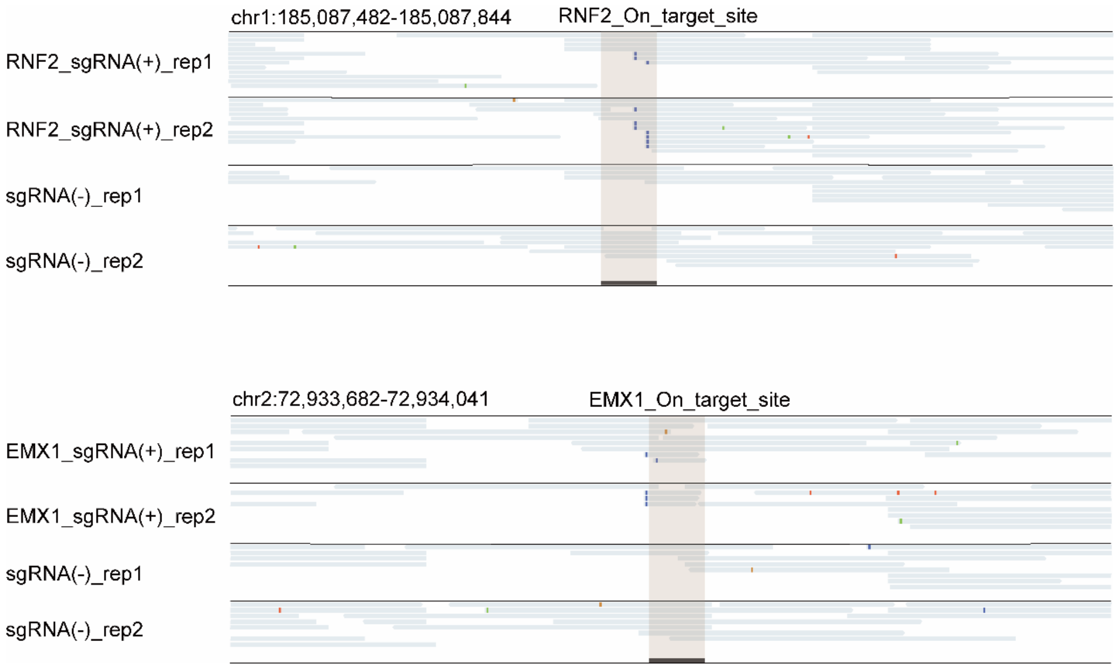

Supplementary Figure S7. IGV views of sequencing data. Sequencing data at the on-target site for RNF2 and EMX1 sgRNA. The editing window is shaded. The orange blocks and blue blocks indicate A-to-G mutations and T-to-C mutations, repectively.

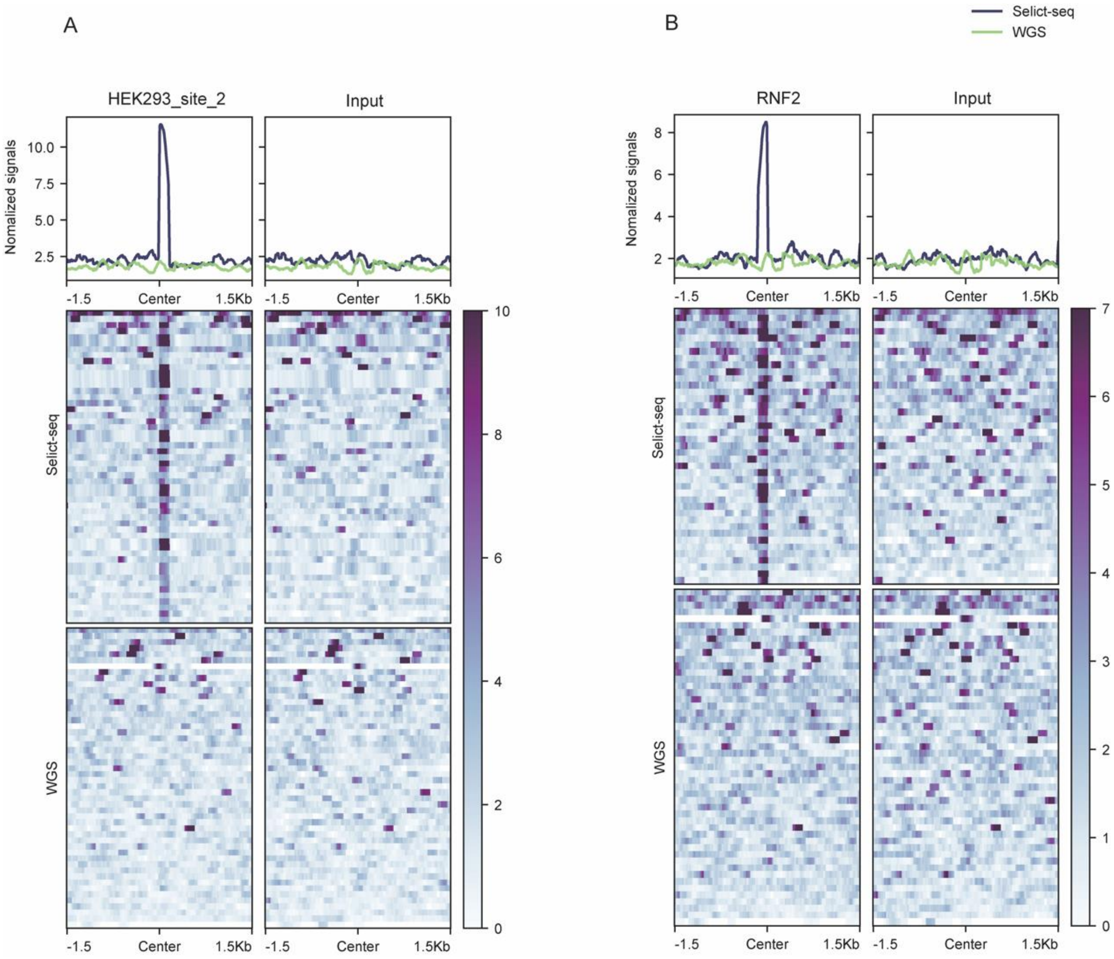

Supplementary Figure S8. Comparison of Selict-seq signals with WGS. (A,B) Heatmaps of normalized signals in WGS and Selict-seq for **(A)** HEK293_site_2 and (**B)** RNF2 sgRNA within 3 kb window. For plots of each sgRNA, the left panel shows signals at the off-target sites in sgRNA(+) samples, and the right panel shows signals in control samples.

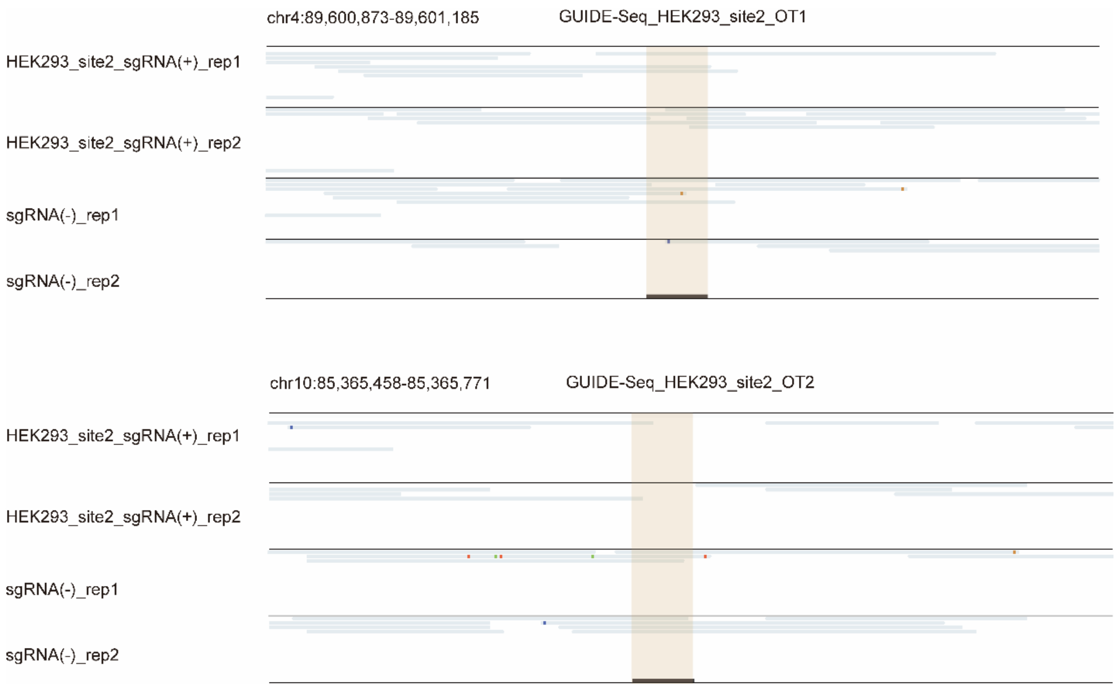

Supplementary Figure S9. IGV views of sequencing data at GUIDE-seq unique off-target sites for HEK293_site_2. Sequencing reads of GUIDE-seq OT-1 and GUIDE-seq OT-2 for HEK293_site_2 sgRNA. The editing window is shaded. The orange blocks and blue blocks respectively indicate A-to-G mutations and T-to-C mutations.

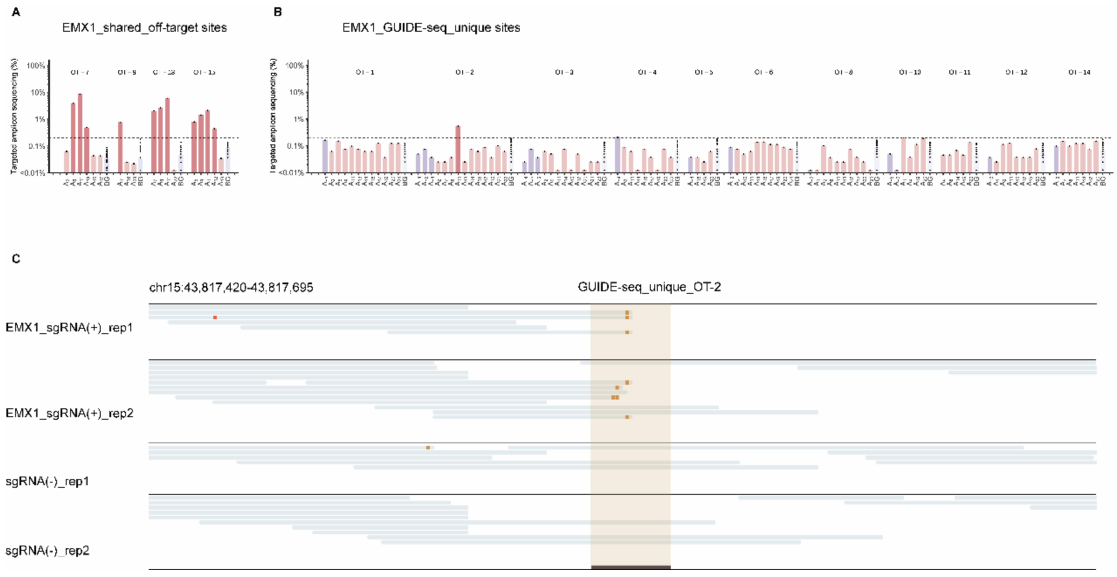

Supplementary Figure S10. Comparisons with GUIDE-seq results for EMX1 sgRNA. (A) Data of targeted amplicon sequencing for the shared off-target sites and (**B)** GUIDE-seq unique off-target sites for EMX1. Adenosines outside the editing window are colored in violet. Adenosines with editing ratios below 0.2% are colored in pink. Background adenosines are shown in gray. (**C)** IGV views of sequencing data for GUIDE-seq unique OT-2. Editing window is shaded and the orange blocks represent A-to-G mutations.

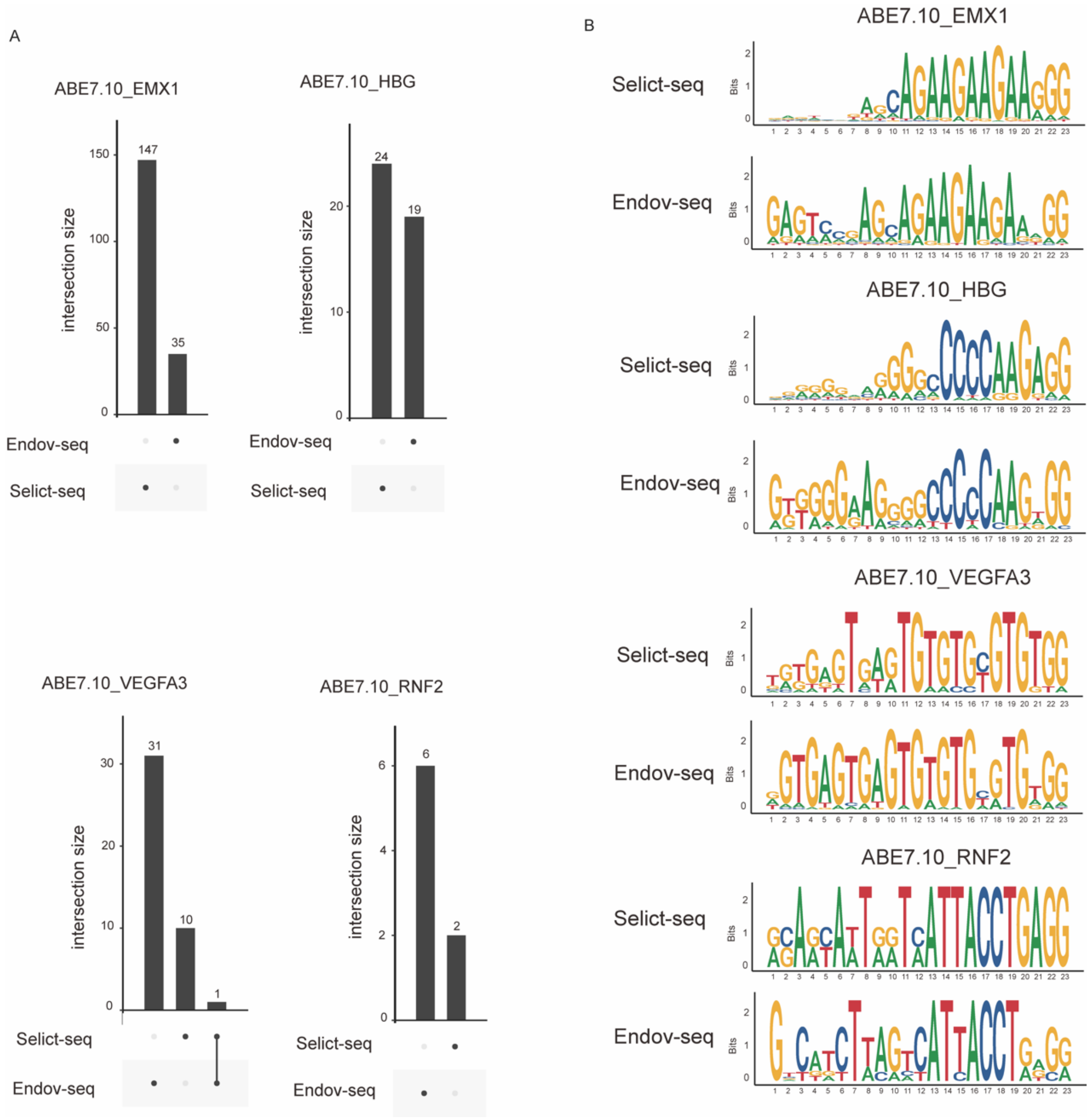

Supplementary Figure S11. Comparison between Selict-seq and EndoV-seq. (**A**) Each plot displays the sites detected by Selict-seq or EndoV-seq for each of the four sgRNAs (EMX1, HBG, VEGFA3 and RNF2). The bar plot shows the number of intersected sites or method-unique sites. (**B**) Sequence logos of Selict-seq and EndoV-seq sites for ABE7.10. Each plot corresponds one of the four sgRNAs.

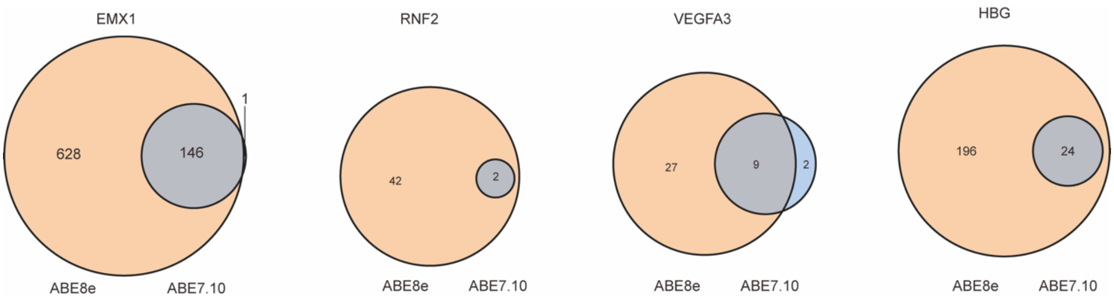

Supplementary Figure S12. Off-target editing analysis of ABE8e and ABE7.10 using Selict-seq. Venn diagrams that compare off-target sites identified by Selict-seq for ABE8e and ABE7.10. Each plot corresponds one of the four sgRNAs (EMX1, RNF2, VEGFA3 and HBG).

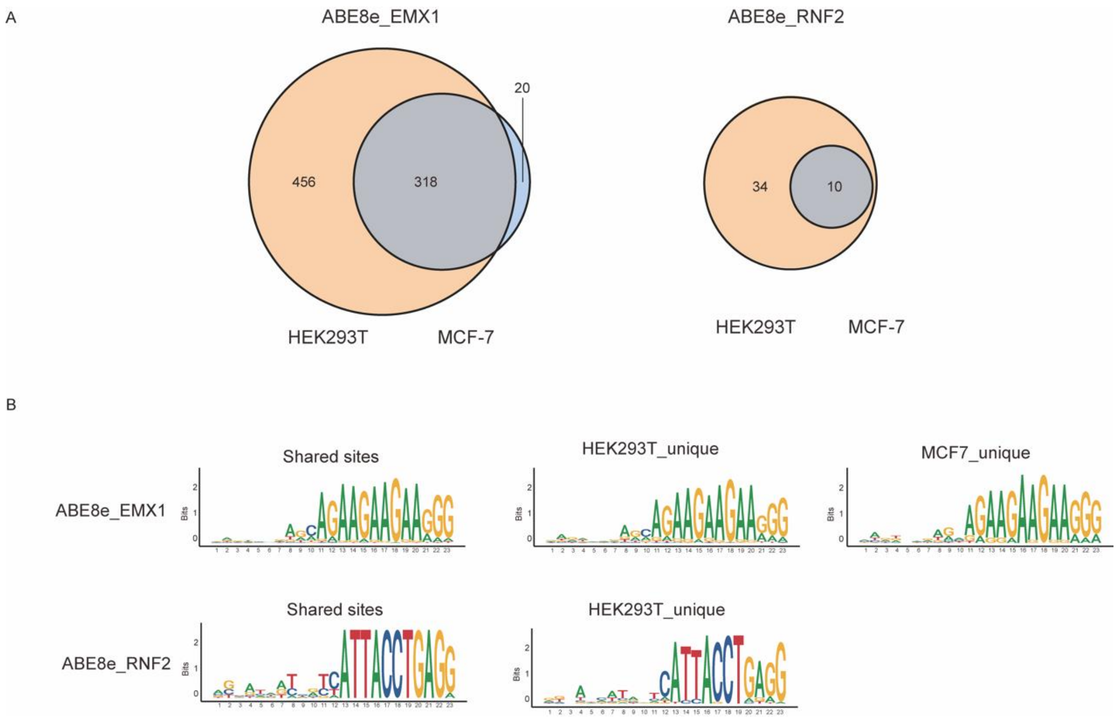

Supplementary Figure S13. (**A**) Venn diagrams that compare off-target sites detected in HEK293T and MCF-7 cells, both edited by ABE8e. (**B**) Sequence logos of off-target sites detected in HEK293T and MCF-7 cells, as well as shared off-target sites in both cell types. Both cell types were edited by ABE8e.

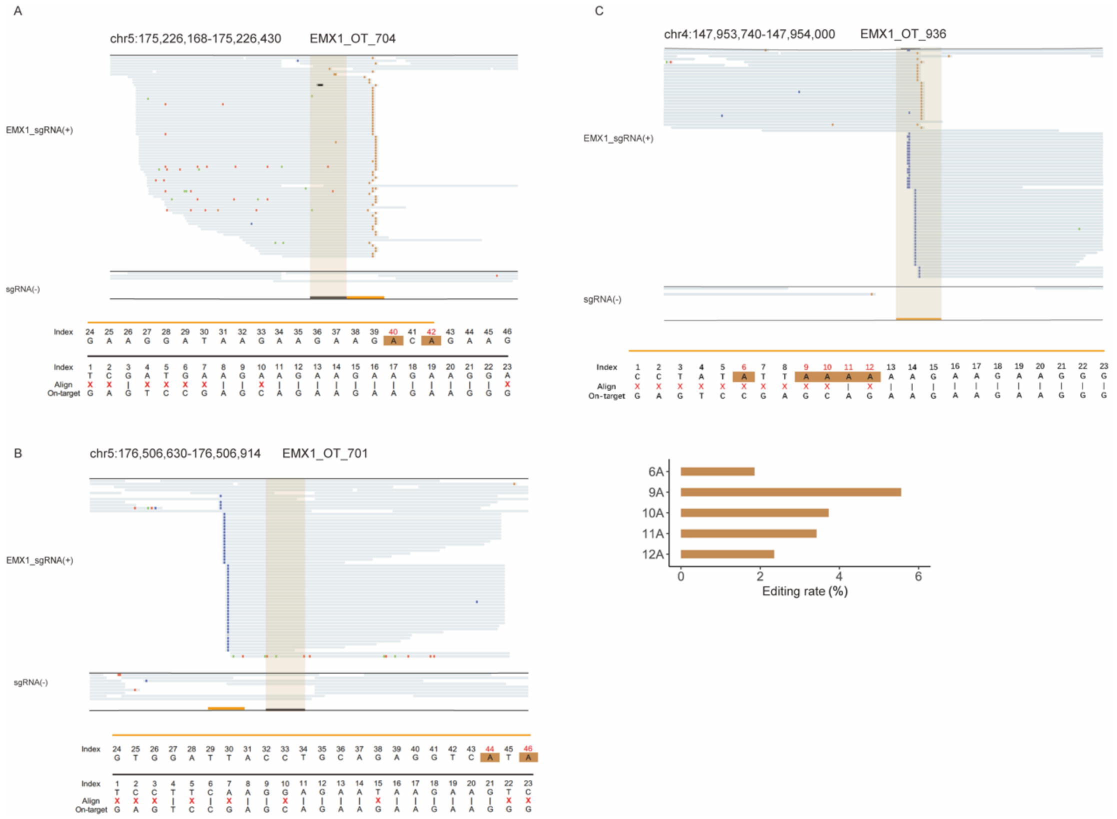

Supplementary Figure S14. Selict-seq discovered out-of-protospacer editing and target-strand editing. (A,B) Representative examples of a out-of-protospacer edit **(A)** and a target-strand edit **(B)**. Both off-target sites locate about 40bp away from the editing window. **(C)** A genomic region with both out-of-protospacer edits and target-strand edits is shown. The editing window is shaded. The orange blocks and blue blocks represent A-to-G mutations on the non-target strand and target-strand, respectively. The corresponding editing rates are shown at the bottom.

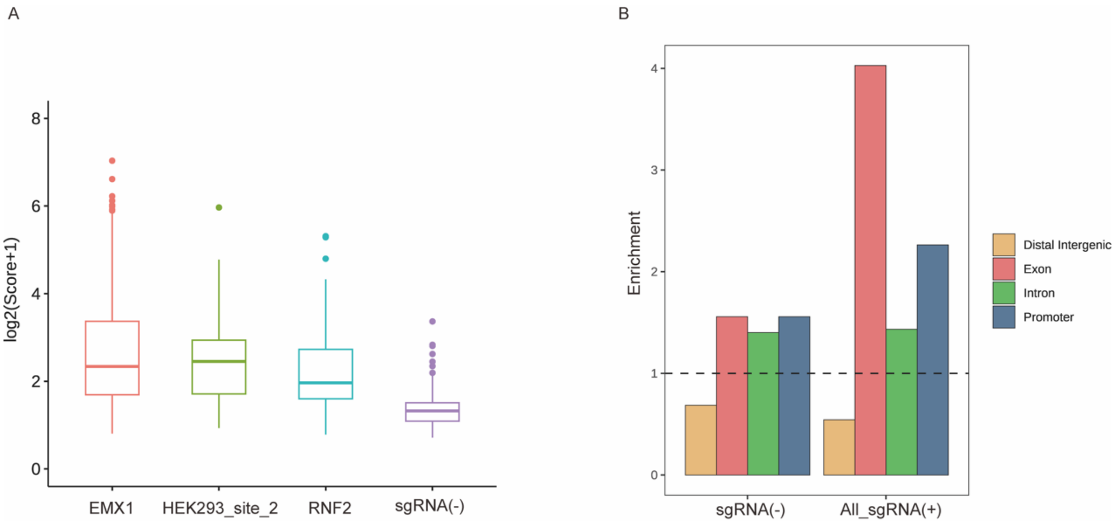

Supplementary Figure S15. Features of the Cas9-independent off-target sites in ABE8e. (**A**) The box plot shows the Selict-seq scores across different samples, with the Selict-seq scores being lower in the sgRNA(-) samples. (**B**) Relative enrichment of sgRNA(-) and All_sgRNA(+) sites are enriched in the gene regions compared with the untreated sampling data.
